# Access to healthcare: a promise fulfilled? A hoax? Or, a matter of control? A qualitative study into the experiences of international students in Hungary

**DOI:** 10.3389/fpubh.2025.1635089

**Published:** 2025-10-23

**Authors:** Livia Yawa Like Atiku, Emmanuel Adofo

**Affiliations:** ^1^Doctoral School of Clinical Medical Sciences, University of Pécs, Pécs, Hungary; ^2^Department of Obstetrics, The Bank Hospital, Cantonments, Accra, Ghana

**Keywords:** access, healthcare, international students, Hungary, challenges, experiences

## Abstract

**Background:**

While several studies have examined issues affecting international students in various settings on a global scale, there is a notable lack of evidence about international students in Hungary, and few or no studies have involved these students to better understand their needs. This study aimed at exploring the perspectives of international students on their self-assessed health status, access to healthcare services, and difficulties encountered in Hungary by distilling existing information on how studying in a foreign country has influenced their mental health and general wellbeing.

**Method:**

The study was conducted to extract useful information relevant to the phenomenon from participants in video-recorded focus group discussions (FGDs). Six (6) FGDs were held, hosting students from two universities. A total of 21 international students were purposively sampled. International students aged between 22 and 38 years got featured [ $\bar{x}$ = 27 years, standard deviation (SD) = 4.322]. In general, questions were asked in relation to participants’ stay in Hungary and their experiences while engaging with clinic and hospital staff. Themes that emerged from discussions were analysed by thematic analysis of the transcripts.

**Results:**

Although a comprehensive health insurance covered international students—Stipendium Hungaricum scholarship holders, some had difficulty accessing healthcare services and paid for medications prescribed to them when they thought otherwise. Other related challenges included linguistic barriers to effective communication with service providers, insensitivity, and a lack of empathy from some care providers toward the plight of migrant students. Additionally, there were negative attitudes from some service providers and a tendency toward cold abandonment, as well as concerns about the host nation’s perceived unpreparedness to receive them. This followed pockets of inconsistencies in the form of rigid bureaucracy, delays in responding to email requests for appointments with general practitioners (GPs), long waiting times, and issues of privacy and confidentiality.

**Conclusion:**

Essentially, participants demonstrated they were aware of the guidelines for hospital consultations with a general practitioner (GP). The majority of international students, especially Stipendium Hungaricum scholarship holders praised the healthcare package that was advanced them. However, access to healthcare, which is central to Sustainable Development Goal 3 (SDG3), may be an illusion for some of these students, as the study revealed several obstacles hindering the smooth flow of processes in their quest for satisfactory healthcare. Therefore, this study emphasizes the need for improved systems (infrastructural and human resource capacity building) for a more efficient service.

## 1 Introduction

The presence of international students has become a global phenomenon. The growing numbers of this category of migrants have, according to available statistics, increased within a space of 9 years (2013 and 2022), irrespective of the ravaging effects of the coronavirus disease (COVID-19) pandemic that disrupted an otherwise busy world (1). Recent reports have highlighted that there were over 6.4 million international students globally in 2021—a 3-fold jump from 2 million in 2000 (2).

In the 2021–2022 academic year, the United Kingdom singularly hosted an astounding 680,000 international students, a figure reflective of a 45% rise over word those recorded 4 years earlier. It has also been confirmed that there is a strong positive relationship between global economic growth and global outbound student mobility. On the flip side, a projection has been made that a decline in global economic growth will occasion a moderate slowdown in the growth of international student flows by 2030, and the continent of Europe has been influential in expediting the short-term mobility of students following the rollout of the European Union’s Erasmus scheme (3). According to the United Nations Department of Economic and Social Affairs, in year 2015, an estimated 244 million international migrants, and significantly more people were said to be moving out of their country of origin (4) for various reasons including schooling abroad, thereby creating an urgent need for stakeholders to engage with migration at all levels to support progress toward achieving global health and development targets (5). As of June 2019, the number of international migrants was projected to be almost 272 million globally (6).

In Hungary, the Stipendium Hungaricum scholarship package opened the door for over 11,000 beneficiaries to pursue and actualize their academic exploits (7). The number of international students in its institutions of higher learning continues to grow in significance within the framework of the Stipendium Hungaricum Programme. Ever since the Tempus Public Foundation initiated its take-off project in 2013, it marked the watershed of a convergence ground for student mobility in Central Europe with a prime objective of scaling up the number of international students in Hungary (7). More than 90 countries have partnered with Hungary from across five continents of the world in crystallizing this goal for mutual benefits (8). Applicants at all levels (bachelor’s, master’s, and doctoral) stand a chance of benefiting from the package in addition to part-time and preparatory courses that entice tens of thousands of students each year. In the face of these huge numbers, proactive steps were aligned with the call from global consultations on migration to expedite discussions on the role of research in supporting evidence-informed health responses connected to migration (9).

### 1.1 Figures and trends of student mobility in Hungary

Overall, the number of international students in Hungary increased from 15,000 to 32,000, and their rate among all students increased from 6.9 to 17% between the academic years 2010–2011 and 2020–2021. By the academic year of 2023–2024, their number increased to 37,000.5 (10). The ratio of all international students in Hungary is 14.4% while the ratio of the same group of students on Stipendium Hungaricum scholarship holders is 4.4%. International students are motivated to study in their host nations for several reasons. First and foremost, they are eager to experience new ways of thinking and acting in their field of study. Next, studying abroad enhances career opportunities by providing improved experiences for future employment in their home country or other nations (11). Additionally, international students often receive a broader and more flexible education than they receive in their home country. Finally, they get the platform to build a stronger, independent base that fosters and affirms a more robust, functional network of friendships from an intercultural point of view (12).

### 1.2 Health security for international students

Wading away from the presence and figures of this group of people in host nations, industry players have deliberated and pushed for the health security of a category of the population that is bedeviled by innumerable health challenges, yet their minority status in host populations further deepens their woes amid the struggle to acculturate and adapt (13). In aligning with global consultations on migration and health, discussions regarding the role of research in supporting evidence-informed health responses connected to migration are to be accelerated (9). In line with this, the increasing number of international students enrolling in Hungarian universities necessitates ensuring world-standard quality services. Theories of adaptation shed light on the role of individual and contextual elements in the acculturation process, which determines whether an individual will adapt or not adapt properly to their new environment (14).

Research provides substantial evidence that there is a significant burden of mental illness among some migrant populations (15). Despite the expansion of the international student population in the United States, for instance, they have remained an underserved group on college campuses for several reasons, including language barriers and diversity and inclusion issues (15, 16). Migrants in general are confronted with exorbitant user fees and do incur a lot of disaffection from healthcare providers who have not been adequately trained to exude an acceptable level of intercultural competency as a spontaneous response to foreigners seeking their services. By virtue of their immigrant status, they stand to struggle for health services due to stereotyping and stigmatization. More horrifying is the observation from empirical sources which assert that no matter how bad the situation is for the entire population, it is much worse for foreign migrants (17). Many barriers to accessing services for international students exist (18). These barriers include language, low income, health insurance burdens, and a lack of social support (19). Again, research reveals that university students in their own country are significantly more satisfied with their social lives than international students (20). Mental health problems are also said to be common in college freshmen, and clearly associated with lower academic functioning (21), buttressing the need for a healthy mental state at all times. Mental health among students is not just a student issue; it is an institutional issue of concern that affects and is affected by every member of that organisation. Therefore, it is critically important to have everyone at the table contribute meaningfully to the agenda (21).

In Hungary, international students in need of healthcare services must follow a series of steps to book an appointment with a general practitioner. Appointments and registration are arranged by email on the website of the facility. International students can also request help from an information desk by calling a designated phone line for an appointment or a possible consultation. Regardless of the chosen pathway, booking an appointment is a prerequisite to using any of the services available (22). Guided by this foreknowledge of student mobility and their overbearing obstacles, we took the initiative to conduct exploratory research on international students in Hungary, our first step towards discovery and problem-solving. Based on the outcomes, further research will be advanced. The conceptual framework best suited for this study was the behavioral model by Andersen (14). This framework is to help conceive a mental representation of access and utilisation of healthcare services. It has its tentacles thrown in three main dimensions—predisposing, enabling, and need factors (23).

Health insurance provides leverage to all members seeking medical consultation. Two main types of insurance packages exist in Hungary: the national health insurance system and private health insurance. The national health insurance covers preventive screening tests, outpatient care in the specialties of internal medicine, dermatology, urology, gynecology among others. Included also are laboratory and diagnostic services, ambulatory services, surgeries, a 24-hour call center for consultation (24). The state heavily subsidizes public health services to reduce costs for patients. The subsidy is manifested in the waived consultation fee with a general practitioner, the average cost of which is estimated around €15–30; consultation with a specialist varying from €30–60, depending on the medical specialty; hospitalization, generally covered by local health insurance but may attract additional fees for services not covered or for single room occupation; and medicines. Prescription drugs are usually subsidised, which considerably reduces costs for patients, with the exact amount to be paid depending on the specific medicine and the health insurance brokered (24).

International students on Stipendium Hungaricum scholarship have a comprehensive health insurance, which is the national health insurance plan accessed by the use of the társadalombiztosítási azonosító jel (TAJ) card (the national health insurance card in Hungary) (25). Contained in a Stipendium Hungaricum document is the provision made for the flagship scholarship program, as outlined in this statement (8). Also on the contract form of the scholarship is the segment of commitment toward health (26).

On admission to the university, Stipendium Hungaricum scholarship holders are presented with a document that informs them about the University of Pécs general practitioner services. With the TAJ card, a student can access healthcare, be it in an emergency room (ER), outpatient department (OPD), or during hospitalization (inpatient care)(27).

Having laid the foundation to ensure the welfare of international students is not jeopardized by any unfortunate incidents, several studies have been conducted on various interests in conformity with the culture of generating empirical evidence through learning, discovery, innovation, and a cutting-edge hallmark of excellence (28). It also projects the image of universities to a grander visibility and reputation. Such timely and necessary actions attract attention from relevant stakeholders and investors in a competitive educational sector. Studies about international students in Hungary have centered on the trends of student mobility alongside domestic students with respect to the numbers and economic growth recorded over specific time frames. In one such study, Vincze and Bacs (29) sounded the alarm that though the presence of international students in Hungary has boosted the nation’s economy, the number of native students continues to decline and therefore, sadly, threatens the future of their representativeness in their own institutions of higher learning. Others have discussed issues related to the acculturation of international students in their new environment, highlighting the need to address deteriorating general and mental health due to poor adjustment. However, they also believe that integrating into the Hungarian culture will ease their tension (13). Some researchers have also made projections of winning over 40,000 international students in the future. Embedded in the findings are opportunities and threats that favor or disadvantage the host country (30). Their findings established that respondents wanted to strategically position themselves for numerous benefits that would enrich their lives, while Wu and Rudnák (31) examined the impact of studying in Hungary and found that the experience was an eye-opener for international students to gain insight into entrepreneurship.

This qualitative study was to explore international students’ self-reported state of health, their access to healthcare services, and their general wellbeing in Hungary through objectively coordinated interactions.

## 2 Methods and materials

### 2.1 Study design

This research adopted an exploratory design to prospect valuable information from the personal experiences of international students to identify the patterns and ideas surrounding their access to healthcare services in their host country. The exploratory approach aimed to provide researchers with a comprehensive understanding of the situation from the perspectives of those who experienced it. As a means of maintaining focus, participants were hosted in the discussion in Pécs city, where participants shared common issues about government-funded healthcare facilities. The exploratory design allowed for a better understanding of topics or areas that had limited information (32).

### 2.2 Study setting

The University of Pécs, located in the city of Pécs, Hungary, boasts of a strong international presence, welcoming many international students. It is one of the oldest and largest higher education institutions in Hungary, offering a wide range of degree programmes, including those taught in English. The university is known for its historical setting, safe environment, and diverse student life. The University of Pécs is one of the most internationalised higher education institutions in Hungary, with a significant number of international students and English-taught programs. It is one of the largest universities in Hungary, with 10 faculties and approximately 20,000 students, including over 4,000 international students. Pécs provides a vibrant student life with cinemas, bars, restaurants, and shopping options. Students also engage in sports and participate in exchange programs. The city boasts of a rich history and culture, featuring architectural landmarks, such as the United Nations Educational, Scientific, and Cultural Organization (UNESCO) World Heritage site.

### 2.3 Sampling and data collection process

Our target population was the community of international students in Hungary (those in institutions of higher learning, specifically the universities) who were willing to share information on their self-assessed health and wellbeing, including experiences with the healthcare system of Hungary. Participants were recruited from social media groups based on their status within the semester, targeting individuals who had rich insights to contribute. We settled on having a smaller group that would be able to delve deeper into the subject matter tabled for discussion.

#### 2.3.1 Inclusion criteria

Students affiliated with a university in Hungary who had an active status within the semester (those who had registered in the university portal, Neptun) and were actively attending lectures and other academic programs, or who had completed their programs a semester earlier.

#### 2.3.2 Exclusion criteria

Students who had a passive status in the semester (those who have not registered in the universal portal, Neptun) and were not attending lectures or participating in any academic programs, and who have completed more than a semester earlier.

We wanted a homogenous group (international students in Hungary) that also possessed some degree of heterogeneity (participants from different countries and cultural backgrounds) for purposes of diversity in a multicultural environment.

We also screened participants to ensure eligibility as outlined in our eligibility criteria.

Participants were briefed on the purpose of the group discussions, the requirement to be committed to the time of the call, and the fact that it was without remuneration.

Prospective participants for focus group discussions (FGDs) were recruited from students’ social media platforms through key members. Once they indicated their zeal to participate, they were contacted by phone calls or text messages to inform them about the study and provide further details required by them, including the commitment to ensuring anonymity. Participants were stratified based on their personal timing for availability. Participation in the study was entirely voluntary and totally unremunerated. A pilot study was carried out with four international students who were not part of the final 21 participants to ensure the validity and reliability of the semi-structured interview guide. The demographic data and the interviews were sequenced brilliantly, eliminating names and other personal details that may offer clues to their identity. Indirect coding was used to tag participants instead of their real names. Discussions were held predominantly in groups of three or four at a time. Participants brought to the table their vast cultures and interpretations of healthcare, lending a holistic view to the subject matter under discussion.

In ensuring an objective study was void of any selection bias, we did the following:

1. First, we considered a sample representative enough of international students who volunteered to participate on their own accord. They were also informed of their right to withdraw from participation at any stage of the process at their own will without coercion.
2. We also followed up on those who wanted to cancel participation for their own personal reasons out of curiosity and offered some words of encouragement on how valuable their contributions could be in influencing decision-making at top managerial positions within the healthcare fraternity.
3. The English language was the medium of communication for the discussions since it was common to all students and was delivered in clear and concise words.
4. Maintaining transparency in the research process by carefully considering the research question, participant selection, data collection, and analysis to avoid systematic errors.
5. Other data sources were used to validate findings, which were the findings of our survey on international students.
6. In ensuring validity and credibility, triangulation of data (the use of more than one data source) and analysis (engaging language experts) were factored in.
7. Member checking, peer debriefing, and reflexivity guarded the researchers to minimize the chances of introducing any form of bias into the study.

Spanning the second to fourth week of January 2023, six focus group discussions were conducted with the aid of a semi-structured interview guide. There were alternating groups of three and four participants for the six groups that researchers described as “mini” groups. Participants in all groups were homogeneous in terms of being international students with similar experiences, apart from age differences. On equal grounds, their contributions were diverse in terms of cultural differences between the regions of origin. All groups entertained some degree of vulnerability by participating in the discussions, except the sixth group, which requested an additional level of anonymity to avoid being recorded. Probing questions from the guide (Table 1) were asked to participants at the start of the discussion, and their responses led to follow-up questions. The researchers drafted the discussion guide based on the study’s topic, its objectives, and research questions. The formal language used was English. Focus group discussions were held online with only the principal researcher and discussants present. Responses to nine main questions framed in the semi-structured interview guide were compiled by the researchers for the analysis.

**Table 1:** Focus group discussion (FGD) questions/interview guide.

S. No.	Focus group discussion questions
1.	Could you please tell us about your health since you arrived in Hungary for school?
2.	How would you compare your current status to what it was when you were in your home country?
3.	Have you had any experience with healthcare facilities here in Hungary?
4.	If yes, how do you perceive it?
5.	Do you have any issues affecting your mental health?
6.	Have you enjoyed any support systems so far?
7.	If yes, which kind of support was it?
8.	Do you have any health-related challenges?
9.	What would you recommend to those in authority regarding healthcare services for international students?

### 2.4 Study participants

This study featured international students in higher institutions of learning in Hungary in discussions with the primary objective of exploring their self-assessed health status, experiences with health services, and their general wellbeing. The exploration was to illuminate the researchers’ understanding of the pertinent sociocultural connotations in participants’ own expression of what they perceive about their health, how they sought health services as a need, and their estimation of the services received.

### 2.5 Ethical considerations

Data were collected using a semi-structured interview guide. Students were assured in both verbal and written communication that their identity and the information they contribute will be held in confidence. The standard protocol of ethical consideration based on the Helsinki declaration was followed to uphold compliance in executing the use of data collection tools and processes on human subjects. This study, conducted in accordance with the Helsinki Declaration, was approved by the Institutional Scientific Research Committee of the University of Pécs (approval number: PTE/61924/2021) and the Hungarian Medical Research Council (ethical approval number: BM/26490-1).

Informed consent was obtained from the research participants by signing a consent form, ensuring their voluntary participation and the confidentiality of their information. Confidentiality was maintained by extracting the visual images of participants and by using codes other than the original names of participants. Links to online participation were disabled to sever access to recordings, notes, and transcripts.

### 2.6 Data analysis

We employed the Jeniffer Attride framework of thematic network analysis of qualitative data. This method of data analysis progresses through a sequence of six iterative steps: familiarization, identifying thematic frames, indexing, charting, mapping, and interpretation. The global theme, access to health services, gave rise to two (2) themes and eight (8) subthemes. Data were analysed using the thematic network analysis framework of Jennifer Attride Stirling (33).

The data analysis process began with the stage of familiarization, which saw the researchers immersing themselves in the data to understand its overall content and identify prime areas of interest. This was achieved through repeated readings of transcripts and field notes, which provided references to guides on how to meticulously make initial impressions and identify interesting observations for what to focus on. Striking patterns were identified and recurring ideas noted. Data content, context, and nuances were highlighted before moving into more detailed analysis. Two linguistic experts were tasked with cross-checking the transcription’s integrity against the audio recordings. A 1-week period was used by the researchers to review notes on qualitative data analysis with the help of a data analyst.

This was followed by the development of a coding style based on the order in which focus group discussions (FGDs) were held. All transcriptions were processed through coding, categorisation, and identification of emerging themes with the aid of NVivo version 12. The stage of identifying thematic frames focused on developing initial codes and categories from the data to be used to categorise and organise the information. We identified recurring phrases, concepts, or ideas frequently appearing in the data. Descriptive labels (initial codes) were developed for all recurring patterns by creating a coding scheme and grouping related codes into broader categories of themes. Inductive and deductive approaches were employed from data-driven sources and theory-driven sources, respectively, to capture themes that emerged from the data or were informed by existing theoretical frameworks. Verbatim quotations from interview transcripts were used to illustrate relevant themes.

In assigning codes to relevant text segments (indexing), we methodically applied the developed codes to the data, creating a structured representation of the information. The coding scheme was used to label and categorise the data. Throughout the process, we observed consistency in coding across different parts of the data for a structurally organised data based on the established theme.

Charting organises coded data into thematic networks. Armed with this knowledge, indexed data were thrown into thematic networks, revealing relationships between different themes. Basic themes were grouped into organizing themes. Eventually, the global theme that encompasses multiple organizing themes was identified. We then created a visual network diagram of interconnected themes showing how they relate.

The stage of visually mapping charted data brought out the relationships between themes. In creating a visual representation of the thematic network, we aimed to make the relationships between themes more apparent. A diagram was developed to map out visual themes, their relationships, and their hierarchical structure by connecting basic, organizing, and global themes. Selected shapes, lines, and other visual elements were used to represent the different themes and their connections, thereby enhancing the visualization of the overall structure of the themes and their relationships within the network.

**Figure 1:**
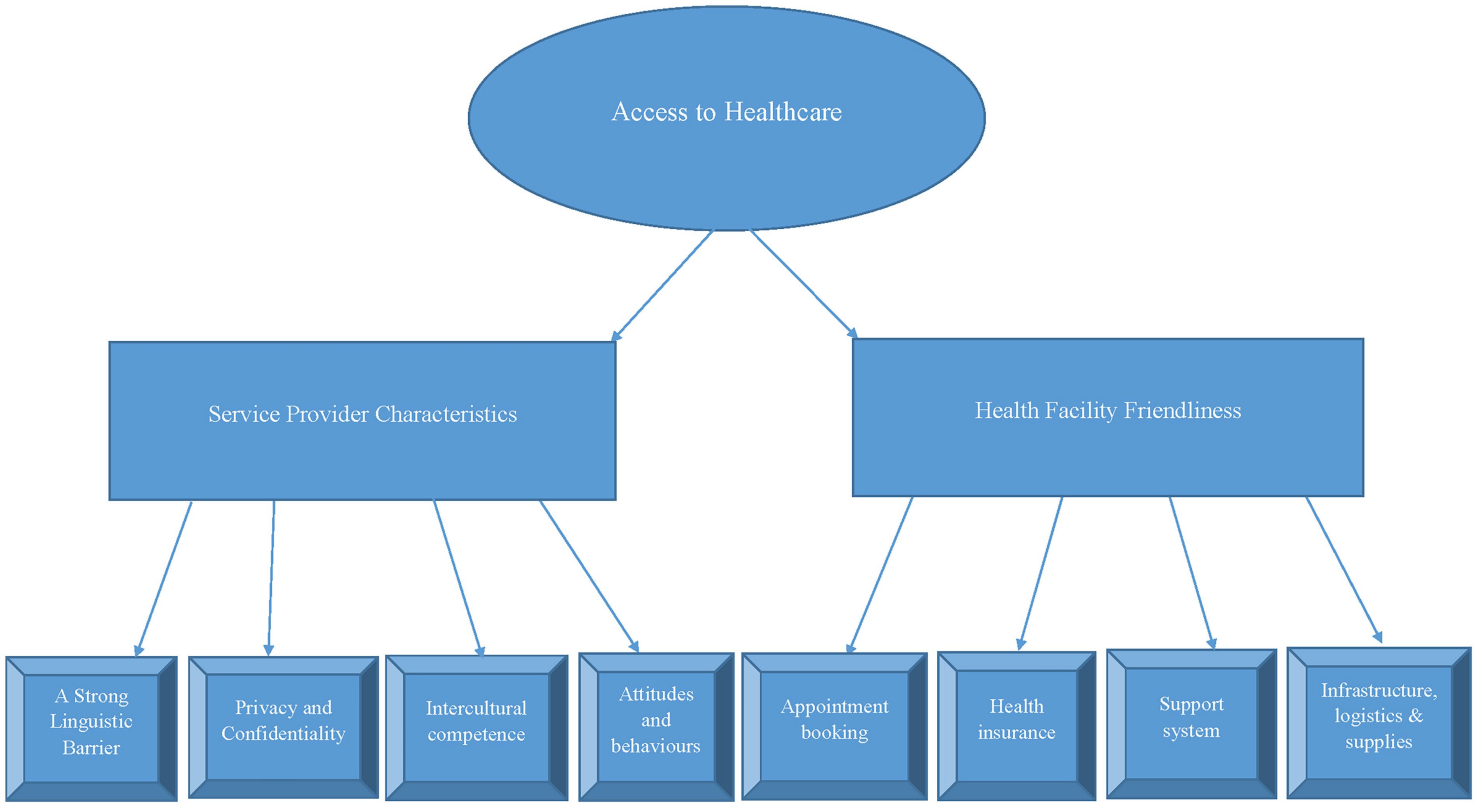
Thematic network analysis using Jennifer Attride Stirling’s framework.

The completion of the mapping stage allowed for interpretations to be made and conclusions drawn to give meaning to and inspire insights from the thematic network. From this, we created our narrative to explain the entire scientific storyline told by the data in the context of the research question and existing literature, and the implications for the research topic. The researchers in this reportage conformed to the consolidated criteria for reporting qualitative research (COREQ) (34) (see Figure 1).

## 3 Results

### 3.1 Sociodemographic data of respondents

Sociodemographic data (Table 2) showcased a spectrum of participants from around the world participating in discussions aimed at exploring international students’ health issues while living in Hungary. For reasons of anonymity, details of participants’ academic engagements were excluded from their demographic data. Predominantly, participants were from the University of Pécs. The gender composition (10 males and 11 females) was indicative of a vocal community ready to exercise their constitutional right of freedom of speech intelligently, void of acrimony, in a very tolerant environment. The majority of participants (85.7%, 18/21) indicated they have had experiences with hospital in Hungary, while the remaining (14.3%, 3/21) have not had any such interaction. The beauty of diversity was elucidated in the richness of different cultural values, age (22–38 years), and gender mix. Again, participants’ age bracket unveiled a blend of youthful exuberance. In the exception of two (2) participants who were self-funding students, all other nineteen (19) were Stipendium Hungaricum scholarship (SHS) students. Fee-paying students funded their health insurance by engaging the services of private health insurance companies, while SHS holders were enrolled in the national health insurance package that provides a comprehensive coverage for all beneficiaries. Countries of origin cascaded from different continents to even include a participant of mixed heritage. This opulent intellectual multiplicity set the tone for an all-inclusive worldview discussion. Apparently, self-funding participants were amazed that their fellow participants were scholarship holders.

**Table 2:** Demographic data on focus group discussion participants.

Serial number	Gender	Age (years)	Country of origin	Institution of study	Educational level	Length of stay (semesters)
FGD/2023/001	Female	(21–25)	Mongolia	University of Pécs	Tertiary	5
FGD/2023/002	Female	(26–30)	Kenya	University of Pécs	Tertiary	2
FGD/2023/003	Male	(32–35)	Ghana	University of Miskolc	Tertiary	4
FGD/2023/004	Female	(21–25)	Nigeria	University of Pécs	Tertiary	2
FGD/2023/005	Male	(21–25)	Kenya	University of Pécs	Tertiary	4
FGD/2023/006	Male	(21–25)	Nigeria	University of Pécs	Tertiary	6
FGD/2023/007	Male	(26–30)	Argentina	University of Pécs	Tertiary	4
FGD/2023/008	Male	(26–30)	Mexico	University of Pécs	Tertiary	3
FGD/2023/009	Female	(21–25)	Iran	University of Pécs	Tertiary	3
FGD/2023/010	Male	(26–30)	Macedonia	University of Pécs	Tertiary	3
FGD/2023/011	Female	(26–30)	Chile	University of Pécs	Tertiary	4
FGD/2023/012	Female	(21–25)	Costa Rica	University of Pécs	Tertiary	2
FGD/2023/013	Male	(26–30)	Israel	University of Pécs	Tertiary	3
FGD/2023/014	Male	(26–30)	Ethiopia	University of Pécs	Tertiary	1
FGD/2023/015	Male	(36–40)	Kenya	University of Pécs	Tertiary	2
FGD/2023/016	Female	(26–30)	Iran	University of Pécs	Tertiary	2
FGD/2023/017	Female	(26–30)	Iran	University of Pécs	Tertiary	3
FGD/2023/018	Female	(21–25)	Mongolia	University of Pécs	Tertiary	1
FGD/2023/019	Male	(36–40)	Syria	University of Pécs	Tertiary	3
FGD/2023/020	Female	(26–30)	Laos	University of Pécs	Tertiary	2
FGD/2023/021	Female	(21–25)	African/Indian	University of Pécs	Tertiary	2

These were their expressions:

“…how did you get it? I do not know about that…I did not know there was.”!! FGD/2023/FBS.

“…that’s great…how do I get to enjoy this too? FGD/2023/FBQ.

Understandably, everyone would like to benefit from Hungary’s benevolent act of lessening the burden of higher education funding for thousands.

Study participants acknowledged a host of factors of diverse degrees and impact were of concern to them, encompassing emotional, psychological, and physical wellbeing. Some had moments to relish from kind native Hungarians, but for the majority of them, there was a thread of mental instability running through the shared accounts associated with culture shock.

### 3.2 Health status on arrival in Hungary

For this question, a good number of six (6/21, 28.5%) participants said they have been well, just as they were in their home country:

“Nothing has changed. I’m okay like I was before.” – Online participant.

Nine (9/21, 42%) reported slight problems adjusting to the cold weather, while the rest (6/21, 28.5%) reported fell sick and required medical interventions.

“I’ve been to hospital many times. I was falling sick often.” – Online participant.

One of such had a different approach to health intervention:

“I tried to take care of myself at home…like making some soup, taking some pills that I have like antibiotics and some pain killers…and I used kind of natural antibiotics like honey, ginger and healthy drinks or different stuff that we can do at home.” – Online participant.

For this segment of the discussion, participants’ responses were categorised into three main segments. There were those who had been in stable health since arriving in Hungary, regardless of the environmental changes, those whose health was disrupted by weather changes, and those who fell sick and required hospital treatment. Interestingly, someone preferred to use traditional means to remedy the situation at home.

### 3.3 Health status comparison

The documented evidence that migration affects health necessitated this question, and responses from participants were intriguing:

“My health status is ok. It is better than before…maybe because I have come to Europe. More milk, new friends.” – Online participant.

For someone, there appears to be a feeling of frustration already, and the best way to reveal it was through a feeling of nostalgia:

“Really, it’s cold even with all these sweaters and winter jackets, I still feel it. Honestly, I miss being home and walking around in simple clothes in a little sunshine. Missing that makes me feel a bit sick…unhealthy.” – Online Participant.

Others admitted the weather in Hungary was cold, but they were not unfamiliar with much more freezing temperatures:

“In my country, we have temperatures as low as −13 °C so this one cannot make me sick. I eat well and drink well. I also wear the right clothing.” – Online participant.

Finally, a note of balance was reached in this response:

“Well, in general like right now, I think I have a stable health.” – Online participant.

In this section, the majority of the participants preferred not to compare their situations for personal reasons, while four of them chose to share their experiences.

### 3.4 Experience with healthcare facilities in Hungary

Speaking on this note, the majority of participants (18/21, 85.7%) have had or attempted to engage the services of healthcare professionals in state-owned hospital facilities. In contrast, a minority of them (3/21, 14.3%) had not, nor had they attempted seeing a physician for various reasons:

“I arrived in Hungary 3 months ago, so I’ve not been to the hospital yet” – Online participant.

Another participant also explained why it has been so:

“If I wanted to go to the hospital, I could. I paid for insurance before because I am a self-fund student, so I needed to do that on my own just to be sure that if there was an accident or anything, so I did that and since I came to university…I take care of myself. I did not go to any hospital…so I did not have any contact with any of the healthcare facilities, yes.” – Online participant.

Traversing a new terrain for orthodox medical interventions, participants revealed there were reliefs and shocks pertaining to the demands of the healthcare system and how proceedings were approached by healthcare professionals. To begin with, access to healthcare services appears to be heavily guarded and probably twisted in the civil and sacred name of bureaucracy for reasons not readily known to participants. The majority of them indicated they encountered some degree of difficulty when they needed healthcare services:

“I contact the general practitioner by email then I got my appointment. It was quite disappointing to me because I sent the email and then they answer like OK, but first you have to take the test and after, if it’s negative twice then we will give you the appointment.” – Online participant.

So much more continued to be said about the entry point and quality of responsiveness:

“And I got appointment from my school hospital, and they just gave me appointment after a few hours, and it was pretty fast too, but I was not satisfied…because I did not get enough information about…my illness and they just gave me a plant-based medicine.” – Online participant.

Participants’ testimonies were given in acknowledgement of what was perceived as good or bad in appreciation or detestation of an act committed or omitted:

“…not like everything has not been good but has not been bad at the same time. I’ve had very good experiences. The bad experience was for some reason I do not understand…why the general practitioners are always recommending this hospital where nobody speaks English. – Online participant.

“Yeah, what’s the motive? What’s behind the scenes? I even asked them if I can go there [400 bed hospital]. They were like “No,” you cannot but then I went, and it did not cost me anything and the doctors are better, and everything is more new.” – Online participant.

Complaints about waiting time were not left out, as patients and clients the world over would cite short waiting time as an indicator of quality healthcare service:

“I had to wait 20 minutes to find a person that can understand my problems and the hospital is like very bad…even the doctor did not want to do his job and he just examined me very quickly and did nothing to help me out.” – Online participant.

Plausible to the discussion was the reality of health insurance coverage:

“When I arrived in Hungary…I realised the Stipendium Hungaricum health funding package covers everything apart from your teeth and eyes…any other thing…be it surgery…” – Online participant.

In a similar manner:

“I still receive the bill for the surgery, and it was an expensive intervention. But fortunately, I did not have to pay it. I had the protection of the TAJ card that allows me to access healthcare in Hungary.” – Online participant.

Hitherto, another participant said:

“They pay my medical insurance from the government, so I do not have to really worry about my medical insurance.” – Online participant.

As nature would have it, the individuality of man will always manifest to break the chain of a particular order:

“The only problem is you as a student, buying drugs and their drugs here are always expensive” – Online participant.

### 3.5 Perception about health facilities in Hungary

This question called for participants’ honest impression about not just physical structures but policies and administrative processes in which services are anchored. The majority of participants who have had access to healthcare services gave pleasant remarks about the services rendered and the resourcefulness of the facility. The liberal opportunity brought along some honest confessions as reflected by this student:

“Comparatively the healthcare is better compared to my country.” – Online participant.

A participant saw things from a different angle:

“I feel like…what do you call it…group ‘B’ students. Yeah, we do not feel like prioritised as the Hungarian students…like so should be all students…same thing. Yeah, I just do not feel like they are very prepared for English programs, and they advertised otherwise.” – Online participant.

### 3.6 Mental health concerns

The realisation that one’s mental health is a conglomerate of their feelings, emotions, and other factors drew participants into the invitation to share their problems related to studies, healthcare, and general wellbeing that have affected their mental health:

“It’s like anxiety all over…studying is hard…like faculty, it’s rough there. They are not very supportive.” – Online participant.

A series of similar sentiments were verbalized:

“Yeah, it’s always anxiety for next semester or next exam, you finish paying and you worry about the next thing to come. Yeah, until we get there.” – Online participant.

Then an expression of what appears like helplessness was voiced:

“We cannot expect much from the school…because they require this stuff and then they are like we do not care. You fail, you fail. They do not have…they will not like… give favour to anyone special.” – Online participant.

The chain of comments kept pouring in, conveying with them the inescapable pressure of a busy semester:

“One of the stressful things about mental health of students was education. A lot of things to do, study Hungarian, study that, do a lot of stuff or perhaps I’ve chosen the wrong country to study in. In my country, we do not take many hours in a semester…nine (9) credits for three (3) courses per semester but the minimum credit hours here are thirty (30).” – Online participant.

Although there was a kind of agreement to the earlier submission, the reaction toward adjusting to the new culture was different based on a previous experience:

“The course work is a lot, but I was used to it before. The course at Bachelor’s was more than this. So, this is better. – Online participant.

Again, some people may have had tough times melting into their new environment, and the discussion provided a chance to let it all out as presented in this voice:

“Yes, yes…I have anxiety at least every day. I came with anxiety before, but it has gotten worse here. It’s like pressures in the faculty…adapting to the new culture, having to be in a hostile environment of Hungarians…and it’s definitely a hard environment to adapt. It’s not for everyone and for someone who comes from a liberal community [we are very smiling people], it is very unwelcoming.

You can feel the cultural shock immediately. – Online participant.

“I have to force myself to go to the hospital because I am afraid that what if they cannot translate into English or what if they do not know about…my full…history about my illnesses, etc., so, it was like kind of middle, I would say the experience was.” – Online participant.

This honesty from one participant confirmed they were not looking for a perfect Hungary. They only wanted a little bit of commitment from Hungarians:

“The city in general, it’s not very prepared to receive this number of international students. I do not expect every Hungarian to know English, I do not expect the lady at the bakery to reply to me in English. But I mean if I go to a doctor’s office, I expect them to speak English. So, it’s a bit weird that they do not have staff that has a reasonable level of English.” – Online participation.

Weighty in this discussion is the dominance of adaptation to the new location that continues to take the center stage:

“I had this problem of being in a new environment…also communication barrier was a problem to me when I came. With some of the attitude people here displayed, it was so alarming for me but as time went on, I adjusted to the whole situation.” – Online participant.

Undoubtedly, the language barrier remains a big hurdle to cross:

“Honestly, I feel like I have to force myself to go to the hospital because I’m afraid…what if they cannot translate into English?” – Online participant.

Typical situations were mentioned as areas deficient in lending support to international students:

“I’m talking more about my faculty in particular the healthcare…they are like I think not prepared for international programs…like I feel we look at the Hungarian students; they are like more accommodated.” – Online participant.

Meanwhile, the University of Pécs has made provision for students’ mental health services. The center addresses their concerns by helping students maintain contact with their environment through various channels, including chats, common events, and transportation. It also assists with the proper treatment for possible mental harm (finding the proper expert, if needed) and preventing crisis situations or treating actual crises. Hitherto, it still may not be a means to an end without an extra measure of expanded services, as depicted in the tone of a participant:

“I cannot get therapy because there’s no psychologist that speaks English here.” – Online participant.

In the dimension of adequacy of the sessions allowed for a student per semester, this concern was raised:

“First, these counselling services are limited to five (5) per semester, so I think they are not enough because mostly they are dealing with personality problems, mental problems or health problems…like be recognised and diagnosed.” – Online participant.

### 3.7 Barriers to access

This segment attracted the patronage of the majority of the participants. As is familiar with every project, the possibility of facing challenges is especially high in its fundamental stages. Being an international student in Hungary is not excluded from this. Discussions on this subject matter brought to light situations that appear to be obstacles in the way of international students attempting to enter health facilities in Hungary:

“As a student, I encountered a challenge when I got sick on Friday and had to wait for an email response on Monday” – Online participant.

Apparently, the protocol of booking an appointment by email is strictly adhered to so that if a student walked to the healthcare facility without doing the needful, they were denied any care service and redirected to book an appointment online or go to the emergency room (ER):

“Once I even went personally to the medical consultation and they told me, like, no, we cannot see you…you must ask for the appointment by email, and I was like, okay, so then what? So, everything must be via email. That’s weird, because when you are sick, you of course need the doctor to see you in person.” – Online participation.

The above expectation may sound as though the participant was asking for too much, but that may not be the case since the idea of out-of-hours services is typically common in countries like Germany, Spain, France, Italy, the United Kingdom, and Sweden. This service offers organised, suitable, synchronised services in response to clients’ calls during times when medical practices and primary care centers are closed.

Strangely, much as that is the road map to follow, response to such emails is allegedly delayed or ignored, leaving the student in a state of helplessness:

“I got an appointment, and I contacted the doctor. She gave an appointment for two months or later. What it means? The doctor in question responded that he has free time after two months and I went simply to the pharmacy…sold certain medication to me and now I’m okay.” – Online participant.

Further discussions attested to this statement in various ways, expressing shock at some instances:

“It’s just that I send like an email requesting a medical consultation, but the only thing they do…just ask for my symptoms and they ask for photos. In that case I have like sore throat, so they said like, OK, send me a photo of your throat and that’s it. So, I sent like the photo of my throat, my symptoms and then they just prescribed my medication.” – Online participant.

Participants consistently verbalized that their experiences were strange:

“I would call them and then they ask me my symptoms, what kind of doctor I need and then tell me, okay so this address and this address are available and this time and this time and then I could go, and everything would be free.” – Online participant.

Some students said the bottlenecks associated with following this protocol are exasperating. For such reasons and more, they resort to some homemade remedies or over-the-counter drugs:

“I heard about how uneasy it can be to get medicine at the pharmacy, unless you have doctor’s prescription.” – Online participant.

Some friends became the lifeline to the deadlines of their colleagues:

“Like if I did not have my partner with me and she wasn’t Hungarian, I would have been scared because every time I got sick here, she was the one who searched for the doctor, find [got] an appointment for me. I just showed up. Sometimes she was on a call with me and she translates what I should have answered. And it was like not a very convenient experience for me.” – Online participant.

A participant lamented over how an exigency had to be personally funded, but thought the service rendered did not merit the fee charged:

“Once I had a problem where I needed to go to the[specialist] clinic and for some reason [the specialist consultation] is not supported by any healthcare so it’s private. You cannot get an examination from a [specialist] doctor for free so I had to pay 35,000Ft…the people at the state hospital and the GP also told me to go to the private clinic so yeah, I had to pay like 35,000Ft just for an exam…just for the doctor to prescribe antibiotics for me which I think should not cost that much.” – Online participant.

All through the discussion, the majority of the participants hammered on access to the facilities as their main hindrance, but this singular perspective made a strong point:

“My opinion is that Hungary has actually good medical services, good access but the only problem is that entry level.” – Online participation.

From the outpour of experiences shared, participants had difficulty securing appointments with GPs. These difficulties, including delayed responses to emails, long waiting times for consultations, high costs for low-value services, and language barriers, have led to the displeasure of international students with the healthcare services they received.

### 3.8 Support systems

International students participating in the study mostly stated that family and friends were their only support systems:

“I have found like also a nice community of Hungarians. I also work with Hungarians which is pretty nice, but it took…at least a year to find the nice Hungarians. it’s definitely a hard environment to adapt.” – Online participant.

The relevance of support systems cannot be underestimated at this time, and building such became a necessity, as echoed in this voice:

“My colleagues in school…that’s the best support…encourage each other and everyone. If someone is giving up, we help them. We get like a help, and you get help also…it’s like we are in this together.” – Online participation.

For someone, help came faster than may have been anticipated:

“I was lucky enough to find a support group here in Hungary quickly.” – Online participant.

The participant did not withhold that innate joy by sharing with others those cordial relations held with a native Hungarian:

“Yes, and in my place of work. I had this lady. She is Hungarian. The woman was so nice to me so each time I come her way, she’s so welcoming.” So, she comes around to check on me. I also go to her place…since then, she became my family.” – Online participant.

As mental health issues continue to advance the discussions, notable acknowledgements in favor of the Hungarian health system were made:

“My general mental health is awesome, and Hungary has good support system for supporting mental [mentally] related issues. The psychiatrists are doing a commendable job in restoring the mental health of patients. This comes from a few students whom I have interacted with and had mental [mentally] related issues.” – Online participant.

Despite the availability of such services, some students are unable to benefit from them due to factors beyond their control. On the issue of seeking counselling to deal with the effects of some of these negative encounters, this was unraveled:

“I heard that there is one psychologist in my university, I had a little bit of mental problems, it’s hard to adapt in the new environment and it gets a little bit harder but…there’s just little time to cope…deal with my mental problems.” – Online participant.

To finalise this section, a participant said:

“Mentors, friends and family” are the core of support.” – Online participation.

Participants spoke extensively about the support group they draw strength from to survive in a foreign land. From their contributions, it can be deduced that life in a host nation is not exactly as in one’s home country. Everyone makes a commitment to another person or reaches a compromise on issues for mutual or collective benefit.

At the point of making recommendations to healthcare authorities for international students, the most striking call was one to avert impending dangers associated with drug administration errors. This participant called for a position on pharmacovigilance and accessible databases of patients’ records for educated referencing:

“I also want to add that the general practitioners, they should be more serious in their work…they gave me medicines that should not mix…they do not have any evidence from your past.” – Online participant.

Based on participants’ feedback about the support they received, they recognise the importance of support systems. They have demonstrated this understanding through personal efforts, playing a complementary role to the host institution’s initiatives by being there for their colleagues and providing support when they needed them.

## 4 Discussion

This study contributes generously to Sustainable Development Goal 3 (SDG-3), ensuring good health and wellbeing for all at all ages. The realisation of this global target has been tied to Universal Health Coverage (UHC) and Primary Healthcare (PHC). Within the context of migration health, international students have been discovered as a minority group in the entire population who are often disadvantaged with a lot of challenges detrimental to their general and mental health. The study explored the self-assessed health status of international students in Hungary, their access to healthcare, and their wellbeing. The motive was to unearth their challenges and implement interventions to remedy them. Plans for the future include having students on stakeholder decision-making boards to serve as the mouthpiece for their fellow migrant students and themselves, and to facilitate the integration of these migrant students into the culture of their host nation by providing safe healthcare services in an inter culturally competent context that will inure to the benefit of both migrants and the entire population within the confines of the destination country.

**Figure 2:**
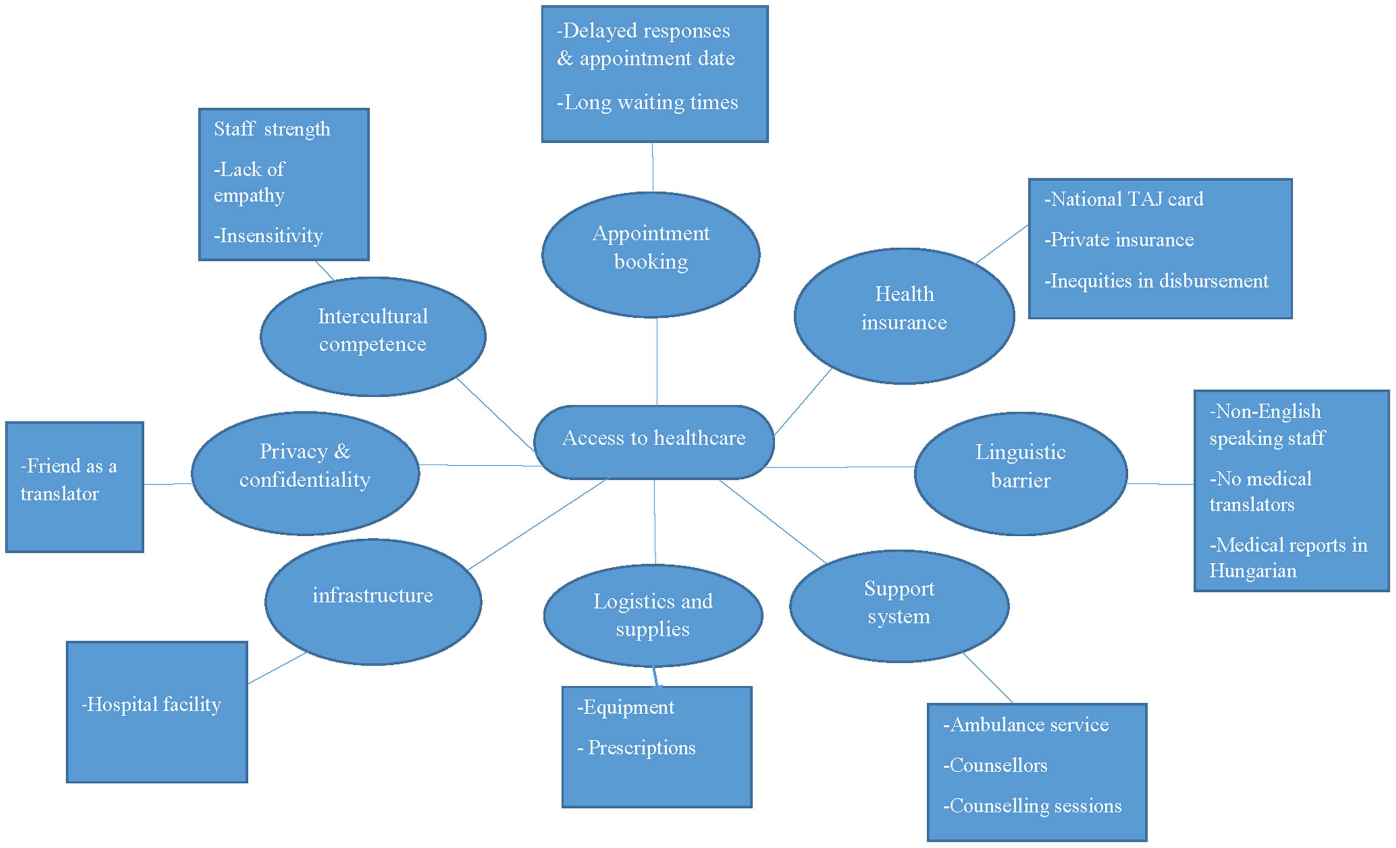
Thematic network analysis.

Asserting that international students face unique challenges, prevention and intervention programs may need to be designed to address their specific problems and prevailing situation. While existing literature has extensively dealt with challenges of international students in host countries (35), this study contributes a wealth of knowledge to the research space by giving an exposition on the concerns of 21 international students of youthful exuberance aged 22–38 years. Although international students appreciate the blueprint for service provision, reviewing the policies guiding it may be necessary since this is the most significant move in shaping how to improve healthcare services to lessen risks for future projects (36). There were similarities in accounts rendered by each participant in their attempts to access healthcare or the reality of having encountered a healthcare service provider. Both sides raised concerns about the authenticity or deficiencies of the services. While there remain many avenues to improving health services for international students in Hungary, this study sheds more light on the unique difficulties hindering access to services and receiving dignified treatment at these service points. In a thematic analysis, qualitative data generated pinpoints several inconsistencies and barriers to effective communication for satisfactory services. This substantiates earlier findings that international students do encounter difficulties when it comes to getting their health needs met (37) (see Figure 2).

### 4.1 Appointment booking and average waiting time

Some of the complaints made in this study stand to supersede arguments that have been advanced in justifying the actions of service providers in the manner in which they were reported. The target of SDG-3 is to improve healthcare delivery to people of all walks of life, irrespective of their geographical location. The essence of Primary Healthcare (PHC) should not be underestimated in the face of vigorous and rigorous global efforts to combat disease and its attendant issues (38). From what participants said, one of their major challenges has to do with being scheduled for timely medical consultations. They felt their attempts had been deeply frustrated by delayed replies to emails pertaining to booking appointments, as well as long waiting times before having a review of their health conditions. This unmet need tends to thwart their efforts at receiving those services they felt entitled to per their understanding of what comprehensive healthcare refers to in their scholarship package (39).

#### 4.1.1 Average waiting time in the Hungarian context

Waiting times for hospital consultations in Hungary vary significantly between the public and private healthcare sectors. Public healthcare is often marked by long waiting lists, potentially exceeding 2 months, whereas the private sector can offer appointments much faster, sometimes within a day. It is even synonymous with it to have a scheduled appointment delayed, with patients potentially waiting 25 min on average for a 13-min consultation.

On the private healthcare divide, appointments are generally much quicker, with some quarters reporting an average waiting time of 8 days for specialist and diagnostic exams, compared to 30 days in the public sector. Patients accessing healthcare at private clinics would normally wait approximately 10 min for their appointment and receive a longer consultation. In the private sector, patients can typically book appointments directly without needing a referral from a general practitioner (GP).

The Hungarian healthcare system is financed through social security contributions that are like taxes. Consequently, Hungarian residents and emigrants employed by a local company, or students on a government scholarship, do not have to pay for their treatment at state-owned healthcare institutions. They use the TAJ card (health insurance card in Hungary) to obtain services at access points. However, there may be costs associated with certain medicines, medical equipment, or extra services when necessary.

The presence of private health facilities provides alternatives to those who cannot endure the long waiting times characteristic of the public system. Benefits of the private system include shorter waiting times and a wider range of specialists within comfortable modern facilities. However, their number is significantly reduced when viewed from the lens of English-speaking services.

#### 4.1.2 Average waiting time in the European context

Waiting times for hospital consultations vary significantly across the organization for Economic Co-operation and Development (OECD) countries. The measurements applied to this variable are therefore likely to use different start and end points. Waiting time can be recorded from the point of the general practitioner (GP) or following a specialist visit to the endpoint of the ultimate treatment where interventions were made (medical or surgical). Waiting times for GP and specialist consultations have been noted to vary more than two-fold across countries (40).

Reports from the 2016 Commonwealth Fund International Health Policy Survey conducted in 11 OECD countries indicated that the majority of people in 2016 were able to get feedback on their medical concerns from their doctor’s office on the very day they contacted the office. The report further stated that in more developed countries like Switzerland, Germany, and The Netherlands, the proportion of people who reported that they “sometimes, rarely or never get an answer from their regular doctor’s office on the same day” was low—12, 13, and 13% respectively—but also higher in countries like Canada and the United States, 33 and 28%, respectively (40).

In the wake of the COVID-19 pandemic, medical professionals were placed under intense pressure to respond swiftly to patients turning up for treatment. The emergency impacted access by affecting consultations in many other specialty areas that had nothing to do with COVID-19. In 2021, the frequency of medical consultations varied widely among the European Union (EU) members. The average number of doctor consultations per inhabitant ranged between 3.5 and 7.8 in the majority of the EU member countries, with the exception of Malta, due to data unavailability (40).

On the topic of medical doctor consultations among EU members, Slovakia presented the highest averages of 11.0 consultations per inhabitant, followed by Germany (9.6), Hungary (9.5), the Netherlands (8.6), and Czechia (7.8), while the lowest were registered in Sweden (2.3 consultations per inhabitant), Greece (2.7), Portugal (3.5), Denmark (3.8), Finland, and Estonia (both 4.1) (41).

Taking the conversation out of Europe, the average wait for a GP appointment in the United States in 2023 was approximately 3 weeks, a time frame perceived to be two to ten times longer than in Europe. On the other side, in that same year, the average waiting time for a medical consultation was 2 days in Switzerland, 6 days in France, and 10 days in the United Kingdom. Comparing the average waiting time in Hungary to that of other EU member countries such as Germany and Switzerland, reveals a significant gap. However, Hungary has outperformed some other countries within the same region. What needs to be done is for Hungary to draw from the tactics of these high performers and tailor them to suit their setting. This report may also confirm the complaints of international students about their experiences with the health sector.

The stalemate in this report suggests that this group of students felt left out due to what may be artificial resistance to their gaining access to healthcare services. Meanwhile, a lack of, or moderated access to, essential health services is an infringement on fundamental human rights as advocated in SDG-3. This finding resonates with literature that states that, though the right to the best achievable standard of health, irrespective of the migrant’s geographical location or status, is protected in numerous human rights policies, national interests do override these entitlements due to the bureaucracies involved (42). Providing skilled attendance for preventive medical screening regularly as a matter of commitment to collaborative efforts of stakeholders is needed at all levels (43). Much as bureaucracy puts systems in check and maintains order, it is also rigid and sometimes stalls the progress of an otherwise functional system (44, 45). Empirically, certain diagnoses cannot be made by simply viewing them in photos. A careful patient history and physical examination is the starting point, and diagnosis over the phone is not advised (46). Viewing from the lens of existing literature, this finding is similar to the work that found that long waiting times for medical consultation are a recipe for dissatisfaction with the service, which may eventually stop people from patronising it in a competitive healthcare world (47). Mounting a defense in favor of Hungary acknowledges the threats of the COVID-19 pandemic at the time, which required two negative confirmation tests for any medical consultations in the country; this is why general practitioners insisted that students comply with their directives. Even with that, other participants with similar concerns have been in the system before the pandemic hit. It is, therefore, more pressing to scale up access to GPs and specialists through systems overhauling. Equipping facilities with the needed human resource training and supplies will allow a smooth flow of care services to this minority group (48). It is surprising to learn that some GPs and specialists were unwilling to examine international students during their moments of need and despair.

Another fact in consonance with prior research studies is the kind of support systems that participants built for themselves. To appreciate the importance of fellow students in surviving outside home is certainly one of their smartest initiatives to strengthen the distant affection they have for family back home. The initiative was aimed to provide emotional and psychological support, enabling students to maintain their focus on their academic engagements. This re-echoes what available research has established that international students rely mostly on friends, family, and a few native allies in their host countries as their most assured support system in order to navigate forward in their academic pursuits (48, 49). This is exemplified in the narratives of participants on how they had to rely on native friends to translate medical information to doctors or fall on them in times of need (50). This finding agrees with studies that have proven language barrier is a palpable obstacle to achieving academic excellence (51). Suggestions from timely interventions have the potential to break the barriers of language non-proficiency of immigrant students, which frequently emerge as the single impediment between countries, stalling the smooth take-off of international programmes (52).

One more concern raised by respondents is the fact that some Stipendium Hungaricum students, although covered by comprehensive national health insurance, pay for drugs prescribed for them by attending physicians in government hospitals. Holistically, despite a lot of successes chalked by the Hungarian health sector, every small deficit remains big as long as it affects human lives and threaten their very existence (53) especially for international students who have to survive in a city with limited student job opportunities to augment their stipends that have not witnessed any upward adjustments (except for PhD candidates) in the face of increased cost for goods and services.

Important to the flurry of discussions is the subject of language minority and immigrant status of students’ social background, factors that shape students’ preparation for higher learning (54). Perhaps, France has set an example that others can look up to and follow. It adopted the most methodical and extensive approach as part of its pedigree, hallmark innovations toward worming its alluring influence into other areas of the world to capture more international students. This massive evolution is being nourished through a matrix of strong structural systems that encompass realistic policies, formidable investments, juicy scholarships, relaxed immigration policies, and effective pedagogic policies for language learning skills to galvanize its position for foreign programs (55).

Igniting the vim to encourage student mobility is an action woven in trusted dedication to reaching future milestones (56). However, integration of international students in general is said to be symptomatically weak due to a lack of adequate and appropriate preparation for staff of the host institution and bias toward African students within the local community of host nations. The level of difficulties with language reported by participants in this study resonates with earlier findings (57, 58). Sociodemographic characteristics, personal beliefs, and institutional track records are but a few to mention that portray such biases (59).

### 4.2 The tussle with acculturation

According to available literature, acculturation to the host culture and to the culture of origin are both adaptive but in different ways (60). Therefore, getting adjusted to new environments after migration is understood as a dynamic process. It may also be said that although international students in Hungary were aware of these changes before embarking on their journey, they were also, to some extent, completely unaware of some of the changes they experienced (61). Evidence supporting this includes statements made by a participant that it took about a year to find natives perceived as “nice Hungarians.” Another example is how one participant perceived the host community as being hostile. While the meaning of the word “hostile” may be generally defined by standard dictionaries, it is also necessary to understand that what constitutes hostility may be relative to the individual. That notwithstanding, the psychological and emotional effects that prompted those statements indicate a kind of worry. Changes such as labeling, stereotyping, humiliation, non-existent moral and physical support, isolation, linguistic barriers, and the like occur in individuals because of negative experiences and become the precursor to mental breakdown. Institutions must make projections as a matter of responsibility to push back the limits and balance the number of students with support services that promote student success (62).

### 4.3 Culture versus standards

Hospital procedures are crafted in both art and science for unique purposes with significance to the human anatomy and physiology. The way and manner these tasks are performed tend to express the values of the people and society at large (63). Participants’ experiences with care professionals revealed their expectations were not met, in contrast to what they were familiar with or even in the scientific lens of physical examination before diagnosis and treatment. This was picked from a statement pinpointing how some GPs attending to international students in Hungary requested patients’ signs and symptoms over the phone and prescribed medications to treat their conditions without laboratory investigations. While it may not be out of place to consider the strategy might have been adopted to guard against the spread of infection during the pandemic, there was no global guideline or protocol that also directed the poor practice. Anthony suggested that such practices, when locally implemented by health professionals, may be borne out of financial constraints in managing a heavy health burden (64). Based on this empirical evidence, the notion that deficient cultural practices (if the allegation was true) could be effectively combined with scientific procedures and regarded as normal is strongly refuted. Researchers have delved into a deeper exploration of cultural elements that influence people and have detected that there could be strings from the Post-Soviet Union states that may have benefited international students from Hungary’s surrounding neighbors, thus giving them a dual experience with Western and Soviet Union approaches to care delivery. Through this, some students could become familiar with some Hungarian traditions related to healthcare and service delivery culture. However, international students from a far distance may take a longer time to acknowledge, understand, and accept the prevailing culture of Hungary (65).

### 4.4 Service quality

The attestation by a participant that healthcare services in Hungary were better than those of their home country credits Hungary for its efforts in care delivery. In another breath, a participant stating that they felt better in Hungary than their home country is in accordance with current literature by Borodina and Pereira, who discovered that international students benefit in multiple avenues socioculturally and economically, among other contexts, by choosing to study abroad (30). Then again, it contradicts what other participants mentioned as factors inhibiting their access to therapeutic care, such as language and mental health support services. This brought into perspective the importance of intercultural competence in the face of a multitude of foreign nationals attracted to Hungary. Vincze and Bács (29) insist improving the facilitator’s language is significant in improving services. Unfortunately, the focus has been on winning more numbers rather than weaving into its gains a necessary training for staff development in areas of culturally competent care (66).

### 4.5 Counselling and support

Oftentimes, it is said that immigrant students do suffer academically compared to their counterparts who are indigenes of their host nation (67). Deducing that what participants raised on mental health are issues that may be considered as ‘normal’ to the flora of migrant student predicaments, some of the concerns affecting them may be negatively impacting their studies. Evidently, the language barrier already presents an impression that native students get better treatment compared to a picture of an assumed neglect in the case of international students is a perfect point of reference in the awakening call to re-assess services being rendered to them. Harboring fears that one’s health problems might not be properly understood during medical consultation is in itself a medical exigency, an unsatisfactory feedback on the services. In a similar study, Marquine and Jimenez (68) observed how cultural and linguistic proficiency in mental health can contribute to crucial professional competency and recommended that they should be incorporated into daily work to improve mental health outcomes.

Al Shamsi, Almutairi, Al Mashrafi, and Al Kalbani (69), prompted that language barriers in healthcare result in misrepresentation of what is being communicated by the patient to the health personnel and what the medical professional perceives the patient to be communicating to them, thus sinking the value of satisfaction on both sides of the communication loop. The quality of healthcare delivery and patient safety is threatened right there in the consulting process (69).

Arguably, some students clearly need therapy to heal from the emotional and psychological trauma they reportedly suffered. Counselling services need to focus more on students’ coping strategies as an efficient way to improve their psychological wellbeing in academic and general life contexts (70). A bolster to this school of thought is the solicitous security-oriented statement of Bal and Perzigian (71), that a state of psychological safety is to be secured through functional systems wired in the basic operations of institutions operated by certified personnel who understand the dynamics of brain activity and have been equipped with the required knowledge and skills for interventions in psychotherapy, and who have also demonstrated competency at their game showing proofs of verified competence.

It is also evident international students may not necessarily have to rely solely on natives of their host country to fully support their interests in a foreign place but through the creation of their own support systems, Recognising and utilising the power in reciprocity to help each other in their lowest moments, they can reap meaningful gains aside a robust formal system (such as guidance and counselling services unit). This melts into the earlier works of Kristina et al. (72), who made an in-depth inquiry into acculturative stress and its associated mental ramifications affecting international students and called for a collective culture that gives individuals the feeling of belongingness to a group whose role of social support will be momentous in curing any maladjustment felt by international students.

A timely caution is sounded to be empirically circumspect in drawing conclusions about immigrants, as their advantages may be overestimated in studies that flaunt a perfectly accurate institutional record rather than a self-reported one from students. This finding conflicts with existing literature suggesting that immigrant advantage may be overrated in studies relying on self-reported rather than school-reported achievement. Participants in this study reported discrepancies between their expectations and the institution’s records, leading to a feeling of being disadvantaged (73).

From a telescopic zoom point and a panoramic point of view, global health education must be incorporated into residency programs in an ethically sound and sustainable manner (74). Recognisably, beautifully outlined in Hungary’s legislation Act CLIV, 1997 on health is its pleasant commitment statements:

“The purpose of this Act is to:

- contribute to ensuring equal access to healthcare services for all members of society,
- create the conditions whereby all patients may preserve their human dignity and identity, and their right of self-determination and all other rights may remain unimpaired.”

### 4.6 Limitations of the study

Participants came from predominantly one university and, therefore, the findings cannot be said to be the exact situation in all other institutions of higher learning in Hungary. In addition to this, the study did not involve other stakeholders. The study was therefore unilateral. Secondly, much of the data was self-reported. Thus, the possibility of introducing some degree of recall bias is subject to the participant’s memory. Transcription of data might have distorted the meaning of what participants relayed in minute details. These notwithstanding, the study offers an exposition on many important lessons.

## 5 Conclusion

Despite the availability of a good number of hospital complexes with state-of-the-art equipment dotting every nook and cranny of Hungary’s beautiful landscape, and the warmth of a crisp professional touch it promises from a high caliber trained staff, there is a strong impression migrant healthcare services for international students still fall short of flexibility and responsiveness as a section of students complained they either had difficulty accessing care or got access but were not handled with compassionate medical care.

Participants also appeared not to have an in-depth understanding of how much entitlement they had under the health insurance coverage. There could be legit reasons accounting for the shortfalls in government hospitals, such as rationing scarce resources to treat an endless influx of demands. However, technically, accelerating the process (doing more within a specific time frame) does not mean it should be violated. That notwithstanding, carers should be mindful of the importance of mental health to this group of people, considering that the majority of them remain young adults and some could even be in late adolescence, as described by some psychologists. The period of adolescence is a difficult one on its own, according to psychologists. Compounding it with migrant stress will certainly complicate matters. Therefore, it will be more profitable for all stakeholders to make commitment the watchword toward their targeted objectives. Otherwise, where there is no commitment, persecution becomes the order of the day, making parties concerned to embark on a fault-finding agenda against each other.

General findings suggest the need for enhanced patient-doctor or patient–nurse relations through platforms like health fora that encourage conversations about health policies, guidelines, and protocols on service access and delivery. General practitioners and specialists doctors, nurses, and administrators who often lead care teams should re-ignite their awareness of culturally sensitive healthcare needs of diverse patients/clients in the light of scientific luminance for a much more humane care. GPs and specialists alike need to pay more attention to pharmacovigilance and develop a personal philosophy to guard their practice in alignment with guidelines on standard practice, as a participant of the study complained a professional prescribed medications not supposed to have been mixed. One fascinating outcome of this study is the point of the paradox when participants’ thoughts that enough preparation was not in place to receive this number of international students was corroborated in the findings of a native researcher recommending that it was time Hungary cut down on admitting more international students for the love of economic gains and took a new tangent toward capacity building in foreign language competency. In summing up, the various sentiments expressed by participants evoked mind-boggling questions as to whether the promises of a comprehensive healthcare stipulated in the scholarship package were real or whether they were a deliberate, appetizing illusion couched to melt the hearts of prospective applicants or to a reasonable understanding, just a structural managerial arrangement to control the mammoth influx of students. Whatever the motive, researchers emphasise the importance of international students being aware and intentional in learning Magyar (a word or two in a day) as they interact with native students, as this is crucial for their own self-efficacy, given that it was essentially their most significant hindrance to acculturation and healthcare. Their self-motivation will complement the efforts of the universities’ compulsory courses in Hungarian for international students.

### 5.1 Recommendations for improvement of IS services

Avenues for intervention to the challenges of international students should be directed at increasing awareness of available services, enhancing communication, and addressing potential language barriers. This means a user can understand the main ideas of complex text and interact with a degree of fluency and spontaneity, making regular interaction with native speakers quite possible. They can also produce clear, detailed text on a wide range of subjects:

1. University-specific information:

Accelerated information and awareness creation can be initiated using clear and accessible guides in multiple languages (English, at least) detailing the Hungarian healthcare system, including how to access both public and private healthcare options. The guide can be included in a brochure for new students on the first day of registration. Considering the number of students who had no idea what their entitlements to health insurance were, it will be expedient for each university to provide clear information on its website and through orientation programs about the specific healthcare services available to international students, including contact information for relevant services and health insurance options. International seasons should include health fairs. Stands can be mounted at such events to open the net for public awareness campaigns that bring health insurance agencies from both public and private sectors. This platform will offer students, who can afford it, a wide range of options to consider for their personal comfort. These platforms could also serve as avenues to educate international students about their rights and responsibilities regarding healthcare in Hungary, with emphasis on the importance of having valid health insurance.

1. Improving access and communication:

This is where intercultural competence displaces cultural competence. Being bilingual or multilingual is an asset. Institutions should shoulder the challenge of expanding their reach to effectively manage the diversity they have invited through their elaborate appraisal of their institutions to the world as choice destinations in the heart of Europe for international programs. Support for efficient services must be garnered toward ensuring access to translation and interpretation services, both at health service centers and through established helplines, to close the gap in language barriers.

Procedures must also be streamlined to simplify the process for international students to register with a general practitioner (GP) and to access specialist care when needed. Accessibility to services must be designed with students in mind. Until they are involved in the decision-making process, their real needs may not be known, and authorities will just be doing a “guesswork.”

1. Addressing specific needs:

The importance of mental health support in universities cannot be overemphasized. The well-known importance of these support services has aided management to resource institutions in helping students find their rhythm and balance in the choice of programmes to read, career placement and guidance, relationship development and management, and many other social, emotional, and psychological matters while living within the confines of the school. Institutional managers need to recognise this and address the unique mental health challenges that international students may face, including providing access to culturally sensitive counselling and support services. Mental health support services must be crafted in humane acts of empathy and a dedication toward their realisation.

1. Recognize insurance clarity:

Universities should provide clear information about the requirements for health insurance for international students, including options for both European Economic Area (EEA) and non-European Economic Area (non-EEA) students.

1. Financial assistance:

Explore options for providing financial assistance or subsidies to international students who may struggle to afford healthcare costs, particularly those from lower socioeconomic backgrounds.

1. User-friendly facilities:

Facility managers must ensure that healthcare facilities are accessible to all students, regardless of their physical abilities or location within Hungary.

1. Continuous improvement:

The installation of feedback mechanisms for immediate verification at the end of service provision will be valuable for monitoring and evaluation processes. Requests for this feedback could be in the form of a short messaging service (SMS) or an email that sends a link after service has been rendered to rate the service and care providers. This will allow international students to key in their healthcare experiences. The data fed into the system can be analysed, and the findings will inform which areas need to be improved. This activity must be one that the facility commits to by consistently resourcing the unit and ensuring its mandate is carried out for the benefit of the people. The quality assurance unit of each service provision centre must be up to the task by consistently monitoring and evaluating the quality-of-care services provided to international students, generating data through surveys and interviews or focus group discussions to discover areas for improvement. Agencies responsible for accreditation should track performance to assess the effectiveness of the services.

1. Collaboration:

While the internationalization of Hungarian institutions of higher learning is applauded, collaboration between them is the quintessential tonic to maintain their resolute stability and excellence among their peers. Fostering collaboration between universities, healthcare providers, and student support services will not only ensure a coordinated and effective approach to healthcare for international students, but also place Hungary in an indomitable category of world class universities rather than a place of a fine alternative to the best.

#### 5.1.1 Contributions of the study to theory and practice

The findings of this study helped identify gaps in targeted services to international students. The discovery shows there are unmet needs for this group of international students. This calls for dialogues at managerial levels. Reviewing the project will improve service delivery and inform which culturally sensitive interventions would be most appropriate to service consumers.

#### 5.1.2 Contributions of the study to theory

1. Levelling off health disparities:

Our exploratory study sheds light on the specific health disparities faced by international students, revealing factors such as language barriers, cultural differences, and under-resourced healthcare systems that fuel these disparities. This revelation can lead to modifications in existing theories about health inequality and the impact of social determinants on health.

1. Stimulating intercultural communication:

This research prepares the ground to explore how ineffective communication negatively impacts the caregiver-patient interaction. Recognising that this simple interaction impacts service utilisation and outcomes contributes to the development of more distinct theories about intercultural communication in healthcare arenas, with emphasis on the importance of cultural competence and tailored communication strategies.

1. Facilitating acculturation and improving mental health:

Since acculturation issues were one of the challenges of study participants, future studies can be focused on investigating the relationship between acculturation stress, mental health, and healthcare-seeking behaviors of international students in Hungary. This can inform theories about the mental health needs of international students and the immediacy with which culturally appropriate mental health interventions can be developed.

#### 5.1.3 Contributions to practice

1. Improved service delivery as the roadmap to expected outcomes:

Interpreting our findings as practical strategies to improve healthcare services for international students means cultivating multilingual resources in capacity development. This should be achieved through in-service training for healthcare providers on cultural sensitivity and incorporating service delivery models into real-time practice to better address the specific needs of this population.

1. Targeted health interventions:

Having identified specific barriers to healthcare for international students, such as financial constraints and the lack of knowledge about the local healthcare system, interventions can be developed to address these challenges. Availing financial assistance to relieve them from the cost of medications and eyeglasses, clear information about healthcare alternatives, or giving seed money to establish peer support networks will be a shared responsibility.

1. Clear channels of communication

The research highlights the importance of effective communication between healthcare providers and international students in Hungary. Implementing strategies such as the use of trained interpreters, providing written materials in multiple languages, and offering cultural sensitivity training to healthcare staff will be a frog-leap to the giant step in unblocking choked communication channels for lifesaving transactions.

1. Increased utilisation of migrant-friendly services:

In removing barriers to healthcare access and improving the overall healthcare experience, exploratory studies can encourage international students in Hungary to seek timely and appropriate healthcare. This can lead to better health outcomes and academic success for this vulnerable population.

### 5.2 Recommendations for future studies and use of data

By identifying potential relationships between variables and formulating specific, testable hypotheses that can be evaluated in a structured or comparative design, new studies can be conducted from the findings of this research.

1. We can also try descriptive studies that quantify the prevalence of barriers to access identified in the exploratory phase and follow up with a comparative study that examines how access differs across demographic groups or geographic regions. More so, as public health experts, intervention studies that test strategies to improve access based on the insights gained from this current study should be greatly considered in:

- comparing different groups at a single point in time (cross-sectional studies),
- tracking the same groups over time to assess changes in access (longitudinal studies), and
- comparing groups with and without a specific health outcome to identify factors associated with access difference (case control studies).

1. Intervention studies may be used to test the effectiveness of interventions designed for improved access based on the findings of the exploratory study.
2. The use of a mixed method approach, that is, quantitative and qualitative methods to complement or consolidate findings.
3. Feeding data sources with new discoveries as documented evidence for reference and advanced studies.

## Data Availability

The original contributions presented in the study are included in the article/supplementary material, further inquiries can be directed to the corresponding author.
